# The disease stage-associated imbalance of Th1/Th2 and Th17/Treg in uterine cervical cancer patients and their recovery with the reduction of tumor burden

**DOI:** 10.1186/s12905-020-00972-0

**Published:** 2020-06-17

**Authors:** Wei Lin, Hua-ling Zhang, Zhao-yuan Niu, Zhen Wang, Yan Kong, Xing-sheng Yang, Fang Yuan

**Affiliations:** 1 Department of Obstetrics and Gynecology, The Affiliated Hospital of Qingdao University, Qingdao, People’s Republic of China; 2 Department of Obstetrics and Gynecology, Qi Lu Hospital of Shandong University, Jinan, People’s Republic of China

**Keywords:** Uterine cervical cancer, Th 17, Treg, Immunologic dissonance

## Abstract

**Background:**

Nearly all uterine cervical cancer (UCC) cases result from human papillomavirus (HPV) infection. After high-risk HPV infection, most HPV infections are naturally cleared by humoral and cell-mediated immune responses. Thus, cervical lesions of only few patients progress into cervical cancer via cervical intraepithelial neoplasia (CIN) and lead to persistent oncogenic HPV infection. This suggests that immunoregulation plays an instrumental role in the carcinogenesis. However, there was a few studies on the relation between the immunologic dissonance and clinical characteristics of UCC patients.

**Method:**

We examined the related immune cells (Th1, Th2, Th17, and Treg cells) by flow cytometric analysis and analyzed their relations with UCC stages, tumor size, differentiation, histology type, lymph node metastases, and vasoinvasion. Next, we quantified the Th1, Th2, Th17, and Treg cells before and after the operation both in UCC and CIN patients.

**Results:**

When compared with stage I patients, decreased levels of circulating Th1 cells and elevated levels of Th2, Th17, and Treg cells were detected in stage II patients. In addition, the imbalance of Th1/Th2 and Th17/Treg cells was related to the tumor size, lymph node metastases, and vasoinvasion. We found that immunological cell levels normalized after the operations. In general, immunological cell levels in CIN patients normalized sooner than in UCC patients.

**Conclusions:**

Our findings suggested that peripheral immunological cell levels reflect the patient’s condition.

## Background

Uterine cervical cancer (UCC) is among the most common malignancies diagnosed and is a leading etiology of malignant tumor deaths in young women worldwide [1]. In many developing countries, it causes more than a quarter of a million deaths annually because of grossly deficient treatments. Human papillomavirus (HPV) infection is a leading cause of uterine cervical cancer worldwide. After high-risk HPV infection, most patients at this time clear naturally as a result of immune responses [2]. Only few cervical lesions progress via cervical intraepithelial neoplasia (CIN) into cervical cancer [3] because of the persistent oncogenic HPV infection [4, 5]. Since numerous cases go through the CIN stage, most UCCs can be clinically detected [6]. Considering the pathogenic factors, immunoregulation probably plays an instrumental role in the HPV-induced carcinogenesis. Some important types of CD4+ cells, such as Th1, Th2, Th17, and Treg cells, have important functions in the pathogenesis of various autoimmune diseases and in mediating host defensive mechanisms against various infections [7–11]. Treg cells are a functionally immunosuppressive subset of T cells, and this vital function is exercised alongside the detrimental effects on tumor immunosurveillance and antitumor immunity [12]. Evidences from cancer patients suggest an association of increased Treg activity with poor immune responses to tumor antigens, thus contributing to immune dysfunction [13]. An imbalance among these T cells will either lead to an immune response or its suppression [14].

The balance between Treg and Th17 cells reportedly controls the immune response and is an instrumental factor in regulating helper T cell function associated with the Th1/Th2 shift in autoimmune diseases and graft versus host disease [15]. In our previous studies [16], we found imbalances of Th1/Th2 and Th17/Treg cells in patients with UCC or CIN. In addition, the situation in UCC patients was more serious than it was in CIN patients. Recently, we measured the levels of Th1, Th2, Th17, and Treg cells in UCC patients at different stages; furthermore, we also measured them before and after the surgery to detect their possible roles and identify the relationship between immune imbalance and uterine cervical cancer progression.

## Methods

### Materials and samples

Seventy-nine fresh specimens of human samples were acquired from the Department of Gynecology, The Affiliated Hospital of Qingdao University. This research was approved by the ethical committee of The Affiliated Hospital of Qingdao University; written informed consent for participation in the study was obtained from each subject. Besides, the research was in compliance with the Helsinki Declaration revised in 2000.

Thirty-eight untreated UCC patients (age range 39–69 years, 46.2 ± 6.9 years) and 21 untreated CIN III patients (age range 25–55 years, 42.3 ± 3.9 years) were enrolled in this study. The characteristics of UCC patients are presented in Table 1. Patients complicated with cardiovascular diseases, hypertension, diabetes, pregnancy, connective tissue diseases, active or chronic infection, endometriosis, or a history of malignant tumor were excluded. No initial immunosuppressive, radiotherapy, or chemotherapy was performed before the surgery. All cases were histologically proven; the clinical stage of UCC patients was based on International Federation of Gynecology and Obstetrics (FIGO) 2009. Twenty healthy women (age range 25–68 years, 42.9 ± 7.1 years) were selected as the control group. The 20 women were all proved to be healthy with normal cervical smear and negative HPV test.

**Table 1:** Clinical characteristics of UCC patients

Characteristic Category	FIGO stage	***n*** = 38 (%)
FIGO stage	I_A_	8 (21.05)
	I_B_	14 (36.84)
	II_A_	10 (26.32)
	II_B_	6 (15.79)
Tumor differentiation	Well	8 (21.05)
	Moderate	14 (36.84)
	Poor	16 (42.11)
Histology type	SCC	32 (84.21)
	ADC	6 (15.79)
Tumor size (cm)	<4	23 (60.53)
	≥4	15 (39.47)
Lymph node metastases	Positive	8 (21.05)
	Negative	30 (78.95)
Vasoinvasion	Positive	7 (18.42)
	Negative	31 (81.58)

*UCC* uterine cervical cancer, *SCC* squamous cell carcinoma, *ADC* adenocarcinoma

### Flow cytometric analysis of Th1, Th2, Th17, and Treg cells

We evaluated intracellular cytokines by flow cytometry to reflect the Th1, Th2, and Th17 cytokine-producing cells. Heparinized peripheral whole blood (200 μL) was added to an equal volume of Roswell Park Memorial Institute 1640 medium and was incubated at 37 °C for 4 h in 5% CO2 conditions. The incubation was in the presence of 25 ng/mL of phorbol myristate acetate (PMA), 1.7 μg/mL monensin, and 1 μg/mL of ionomycin (all from Alexis Biochemicals, San Diego, CA). Ionomycin and PMA are T-cell-activating agents that mimic T-cell receptor complex-generated signals and present the advantage of stimulating T cells of any antigen specificity. Monensin was used to block intracellular transport mechanisms, thus leading to cytokine accumulation in the cells. The cells were stained with PE- conjugated anti-γ-IFN, anti-IL17, anti-IL-4, and anti-CD4-FITC (Caltag Laboratories, Burlingame, CA, USA) after incubation. Then, isotype controls were given to enable correct compensation and confirm antibody specificity. The stained cells were subjected to flow cytometric analysis using a FACS-CAN cytometer equipped with CellQuest software (BD Bioscience Pharmingen, San Diego, CA).

Flow cytometry was used to enumerate circulating CD4+/CD25+/FoxP3+ Tregs. Peripheral blood mononuclear cells (PBMCs) were incubated with anti-CD4-FITC and anti-CD25-PC5 mAbs (Beckman Coulter, Immunotech, France) at 4 °C for 30 min. After washing with PBS, PBMCs were fixed and permeabilized with a fixation/permeabilization buffer for 30 min at 4 °C. Then, they were washed with the permeabilization buffer twice and stained with anti-human FoxP3-PE mAb according to the manufacturer’s instructions (eBioscience, San Diego, CA, USA). After 30-min incubation at 4 °C, the cells were washed and analyzed by flow cytometry in a Coulter Epics IV Cytometer (Beckman Coulter, Inc., Fullerton, CA, USA) using Expo32 Software (Beckman Coulter). The cells were gated on viable lymphocytes following standard forward and sideways scattering parameters. Among the cells included in this gating, we evaluated Treg subpopulations as the CD4+/CD25+/FoxP3+ subset. The results are expressed as percentage of triple-positive cells proportion of the autofluorescence of CD4+ cells.

### Statistical analysis

The results were presented as means ± standard deviation(S.D.). The associations between parameters among different groups were assessed using either t-test or one-way analysis of variance. Pearson correlation was used to identify the relation between tumor size and T cell percentage. Generally, *P* values < 0.05 indicated statistical significance. SPSS 17.0 software was used for statistical analyses (SPSS Inc., Chicago, IL).

## Results

### Decreased circulating Th1 cells and elevated Th2 cells in patients with UCC in different stages

**Fig. 1:**
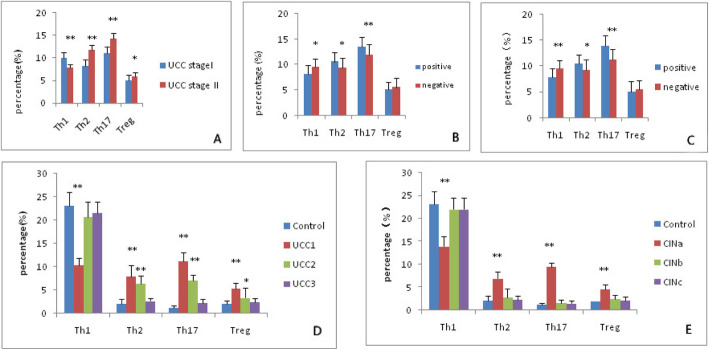
The immune cells related to stages, lymph node metastases, vasoinvasion, and operation. **a** Circulating Th1, Th2, Th17, and Treg cells in patients with stage I and II; **b** The levels of four cell types in lymph node metastasis-positive and -negative patients; **c** The levels of four cell types in vasoinvasion-positive and -negative patients; **d** UCC 1: Testing the cell in the UCC patients before the operation. UCC2: Testing again 1 month after the operation. UCC3: Testing 6 months after the operation; **e** CINa: Testing the cell before the operation. CINb: Testing 1 month after the operation. CINc: Testing 6 months after the operation. Compared to the control groups, *: 0.01<P<0.05, **: *P* ≤ 0.01

We first analyzed the expression of Th1 and Th2 cells based on the cytokine patterns after in vitro activation by PMA/ionomycin in short-term cultures. Among the 38 UCC patients, 22 belonged to stage I, and the percentage of Th1 cells was 10.06 ± 1.24%. The other 16 patients belonged to stage II, and the percentage of Th1 cells was 7.77 ± 0.8%. Compared with healthy controls, a lower proportion of Th1 cells was seen in patients with UCC; the higher was the stage they belonged to, the lower was their Th1 cell percentage (*P* < 0.001) (Fig. 1a). The percentage of Th2 cells in UCC patients was 8.28 ± 1.44% at stage I but 11.82 ± 1.07% at stage II. The percentage of Th2 cells increased with higher stages, and the difference between the two groups was significant (*P* < 0.001) (Fig. 1a). Remarkably, Th1/Th2 imbalance was observed in UCC patients.

### Elevated circulating Th17 cells and Treg cells in patients with UCC at different stages

Compared with healthy controls, Th17 cells were increased in UCC patients, particularly at stage II, as showed in Fig. 1a. The percentage of Th17 cells in patients was 11.11 ± 1.46% at stage I and 14.21 ± 1.37% at stage II (*P* < 0.001). The percentage of Treg cells in UCC patients was 5.02 ± 1.21% at stage I and 5.88 ± 0.84% at stage II (*P = 0.019*). The percentage of Treg cells increased marginally in patients at stage II compared with stage I. The differences of Th17 and Treg cell percentages between the two groups were all significant (Fig. 1a). An evident imbalance of Th17/Treg was also observed in UCC patients.

### The percentages of Th1, Th2, Th17, and Treg cells were associated with the tumor size, lymph node metastases, and vasoinvasion

**Fig. 2:**
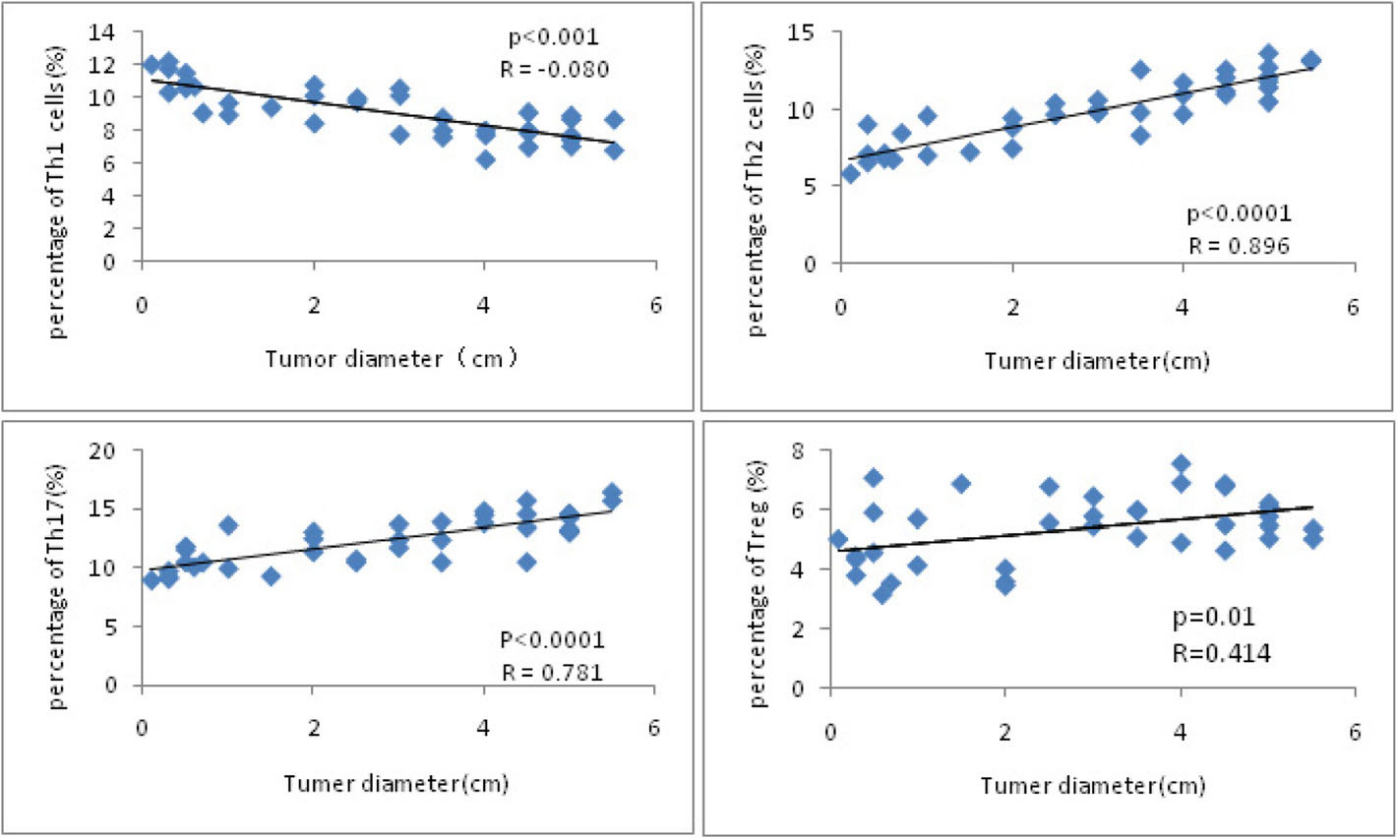
The Th1, Th2, Th17, and Treg cells related to UCC size. The Pearson analysis showed the negative correlation between the Th1 cell percentage in the serum and the tumor diameter (*R* = − 0.080, *P* < 0.001), positive correlation between the percentage of Th2 cells and the tumor diameter (*R* = 0.896, *P* < 0.0001), positive correlation between the percentage of Th17 cells and the tumor diameter (*R* = 0.781, *P* < 0.0001) and positive correlation between the percentage of Treg cells and the tumor diameter (*R* = 0.414, *P* = 0.01)

Tumor size was expressed as the diameter measured by pathologists. In Fig. 2, we used horizontal coordinates to represent the tumor diameter and vertical coordinates to represent the cell percentage. The figure showed a negative correlation between Th1 cell percentage in the serum and the tumor diameter. The positive correlation existed between the tumor diameter and percentage of Th2 cells, Th17 cells, and Treg cells.

Among the 38 UCC patients, 30 patients had no lymph node metastases and eight patients had lymph node metastases. Thus, we accordingly separated these patients into positive and negative groups. As shown in Fig. 1b, the percentage of Th1 cells was 8.07 ± 1.80% in the positive group and 9.51 ± 1.59% in the negative group (*P = 0.013*). The percentage of Th2 cells was 10.56 ± 1.81% in the positive group and 9.33 ± 2.01% in the negative group (*P = 0.025*). The percentage of Th17 cells was 13.50 ± 1.93% in the positive group and 11.93 ± 2.10% in the negative group (*P = 0.010*). The percentage of Treg cells was 5.10 ± 1.44% in the positive group and 5.48 ± 1.83% in the negative group. Only the difference of Treg cells between the two groups was not significant (*P = 0.151*).

Thirty-one UCC patients had no vasoinvasion, and seven cases were detected as having vasoinvasion. Thus, we accordingly separated the patients into positive and negative groups. As shown in Fig. 1c, the percentage of Th1 cells was 7.79 ± 1.74% in the positive group and 9.53 ± 1.62% in the negative group (*P = 0.009*). The percentage of Th2 cells was 10.50 ± 1.72% in the positive group and 9.25 ± 2.06% in the negative group (*P = 0.034*). The percentage of Th17 cells was 13.90 ± 2.04% in the positive group and 11.21 ± 2.14% in the negative group (*P = 0.005*). The percentage of Treg cells was 5.03 ± 2.11% in the positive group and 5.52 ± 1.78% in the negative group. Only the difference of Treg cells between the two groups was not significant (*P = 0.252*).

### The balance of Th1/Th2 and Th17/Treg cells recovered after radical hysterectomy in UCC patients

**Fig. 3:**
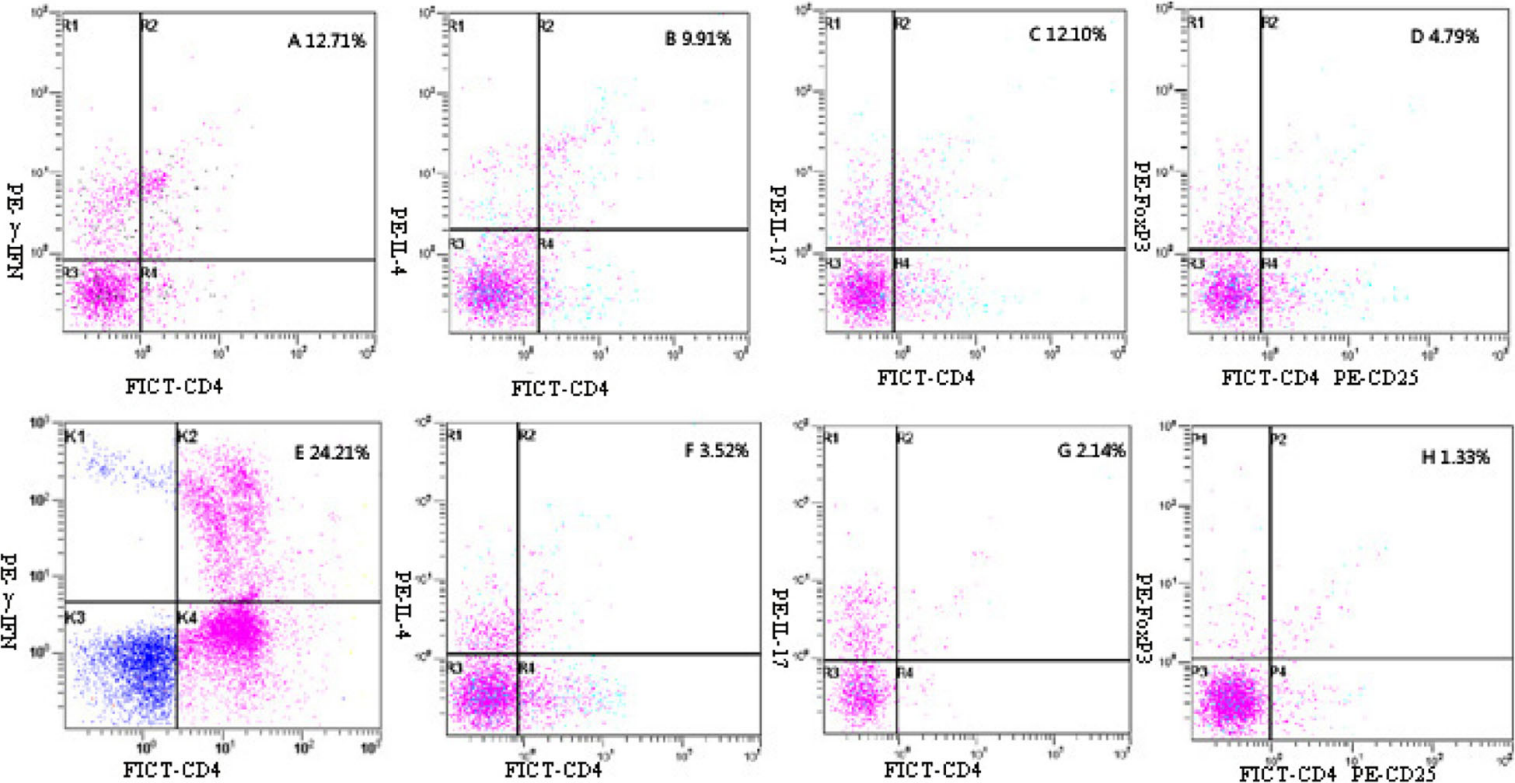
Th1, Th2, Th17 and Treg cells before and after operation in UCC patients. All the cells were stained with PE- conjugated anti-γ-IFN, anti-IL-4, anti-IL17, anti-FoxP3+ and anti-CD4-FITC. Stained cells were analyzed by flow cytometry analysis using a FACS-CAN cytometer equipped with Cell Quest software. The proportions of each cell type in representative UCC patients are annotated in the figure. The proportions before the operations were **a**, **b**, **c**, and **d**. The proportions when tested six months after the operation were **e**, **f**, **g**, and **h**. Th1 cells: **a**, **e**; Th2 cells: **b**, **f**; Th17 cells: **c**, **g**; Treg cells: **d**, **h**. The differences of Th1, Th2, Th17 and Treg cell proportions before and after the operations were all significant (*P<0.001*)

To understand the relationship of the balance of Th1/Th2 and Th17/Treg cells with our therapy, we estimated Th1, Th2, Th17, and Treg cell levels with or without therapy. Patients with UCC had a lower proportion of Th1 cells (10.27 ± 1.51%) compared with healthy controls (23.12 ± 2.81%) (*P<0.001*). However, after the operation, the proportion of Th1 cells recovered quickly 1 month later (20.69 ± 3.19%) (*P = 0.092*) and at 6 months (21.56 ± 2.39%) (*P = 0.055*) (Fig. 1d, Fig. 3). The percentage of Th2 cells in UCC patients also recovered at 6 months after the operation (2.67 ± 0.56%) (*P = 0.309*). However, at 1 month after the operation, the Th2 cell percentage was still significantly higher in UCC patients (6.34 ± 1.76%) than in the control group (2.11 ± 0.99%) (*P < 0.001*) (Fig. 1d, Fig. 3).

Compared with controls (1.23 ± 0.41%), patients with UCC had an evidently higher proportion of Th17 cells (11.25 ± 1.77%) (*P<0.001*). After the operation, Th17 cells recovered at 6 months (2.27 ± 0.81%) (*P = 0.056*). One month postoperatively, Th17 cells had decreased to (7.07 ± 1.19%) (*P<0.001*) (Fig. 1d). The percentage of Treg cells in UCC patients also recovered at 6 months after the operation (2.37 ± 0.89%) (*P = 0.153*). However, at 1 month postoperatively, the Treg cell percentage was still evidently higher in UCC patients (3.34 ± 2.07%)(*P = 0.025*) than in the control group (Fig. 1d, Fig. 3).

We found the balances began to recover at 1 month postoperatively and almost reach normal levels at 6 months postoperatively.

### The balance of Th1/Th2 and Th17/Treg cells recovered after cervical conization in CIN patients

Among the 61 CIN patients, only 21 patients had CIN III and were treated with cold knife conization. To understand the relationship of cervical conization with the balance of Th1/Th2 and Th17/Treg cells, we estimated Th1, Th2, Th17, and Treg cell levels before and after the operation. CIN III Patients had a lower proportion of Th1 cells (13.94 ± 2.11%) compared with controls (23.12 ± 2.81%) (*P<0.001*). However, after conization, the proportion of Th1 cells recovered quickly at 1 month later (22.01 ± 2.5%) (*P = 0.073*) (Fig. 1e). Moreover, the percentage of Th2 cells also recovered quickly at 1 month later in CIN III patients (2.76 ± 1.90%) (*P = 0.058*) (Fig. 1e).

Compared with controls (1.23 ± 0.41%), CIN III patients had a significantly higher proportion of Th17 cells (9.49 ± 0.93%) (*P<0.001*). After the operation, Th17 cells recovered at 6 months (1.49 ± 0.52%) (*P = 0.391*). At 1 month, the proportion of Th17 had already deceased to 1.61 ± 0.69%. The percentage of Treg cells in CIN III patients also reached normal levels at 6 months after the operation (2.18 ± 0.71%) (*P = 0.205*) (Fig. 1e). We found the balances were almost normal at just 1 month postoperatively.

## Discussion

HPV is the most important factor in the pathogenesis of UCC and CIN [17]. Immune imbalance not only influences HPV clearance but also helps cancer cells escape immunological surveillance [18]. CD4+ T-cell suppression or dysfunction has been reported as the mechanism causing cancer escape [19, 20]. Among the CD4+ T cells, Treg cells play a significant role in cancer immune evasion by blocking the induction of immune response against tumor antigens in the periphery as well as by neutralizing tumor-infiltrating effector T cells. The levels of Treg cells are reportedly increased in cancer patients, and their high numbers are associated with poor survival [19, 21]. The balance between Treg cell- and Th17 cell-controlled immune response was a key factor in regulating T helper cell function associated with the Th1/Th2 shift in autoimmune disease and graft versus host disease [15]. In our pervious study [16], we systematically determined that the proportion of Th2, Th17, and Treg cells in PBMCs and of their related cytokines IL-4, IL-10, IL-17, IL-23, and TGF-βI in the serum were markedly increased in UCC and CIN patients. However, serum INF-γ as well as the Th1 cells in PBMCs prominently decreased in UCC and CIN patients. It was verified that Th1*/*Th2 shift and Th17/Treg shift existed in patients with uterine cervical cancer, and these shifts may start from the CIN stage. In another study, Foxp3 positive cells (Treg cells) were also detected higher in SCC group than in the CIN group [22]. The immune imbalance was also detected as the ratio of CD4+ T-cells to Foxp3 positive cells and that of CD8+ T-cells to Foxp3 positive cells were significantly reduced in the SCC group compared to the CIN group [22]. The differentiation change within T cell subsets might contribute to immune tolerance, which helped the cancer cells escape with Treg cells upregulation.

To find the relationship between tumor-related CD4+ T cells and clinicopathological features of cervical cancer, we designed our experiment. We analyzed the four types of CD4+ T cell proportions among UCC patients with different stages, different tumor differentiations and histology types; further, we analyzed the relationship between the four types of T cell frequencies and tumor vasoinvasion or lymph node metastases. We found that the more serious the disease was, the more obvious the changes of the four types of CD4 + T cells were. The cell percentages were all related to the stages, the situation of tumor vasoinvasion and lymph node metastasis. As the disease progressed, the Th1/Th2 ratio decreased as the Th1 decreased and Th2 increased dramatically. Although Treg cells showed upregulation, Th17/Treg ratio also increased because of the marked increase of Th17.

We demonstrated the seriousness of disease was related to the four types of CD4+ T cell proportions in peripheral blood by flow cytometry analysis. Some reports emphasized that increased levels of Tregs were also detected at the cervical tumor site and in the lymph nodes of patients with cervical cancer [23, 24]. The increasement of Treg cells was related to an immunosuppressive status and was proved to be associated with a high death hazard and reduced survival of cancer patients [19]. The large number of Tregs in HPV-derived lesions suggests a pivotal role of Tregs for counteracting the host immune response. In the future, Treg cells might be a target for immune therapy of uterine cervical cancer and CIN [24].

To verify whether immunological CD4+ T cell changes were really related to the tumor burden, we monitored Th1, Th2, Th17, and Treg cell levels before and after the operation in UCC or CIN patients. We found that immunological cell levels began normalize after the operations. It was detected that Th1 cell in UCC group was the first type to recover to normal levels at 1 month after the operation, and then the other three types of cells all normalized 5 months later (Fig. 1d). But in CIN patients, the levels of the four types of cells could all normalize when tested at 1 month after the operation (Fig. 1e). In general, the levels of immunological cells normalized sooner in CIN patients than in UCC patients. The gap of immunological cell levels was larger between the UCC group and the control group than that between the CIN and the control group. Recently, some studies [25, 26] also reported the Th17/Treg balance was broken in cervical cancer patients and the imbalance of Th17/Treg might be involved in the development and progression of cancer. Our findings revealed the treatment really restored the balances.

Previous studies of other cancers [27–29] also showed that Treg cells had markedly higher proportions within PBMCs and that the prevalence of Treg cells significantly differed between early and advanced disease stages. In the current study as well, statistically significant difference of Treg cell levels was found among different stages in UCC patients. Bais found [30] that HPV-infected patients first endured the up-regulation of cytokines secreted by both Th1 and Th2, and then, the Th1 cells decreased and the Th2 cells increased, and finally, the balance was broken. We monitored the Th1, Th2, Th17, and Treg cells before and after the operation and speculate that the Th1 cells may experience the same situation as Th1 cells recovered first after the surgery and the balance was then restored. Persistent HPV infection can lead to immunologic derangement, and immunologic dissonance helps cancer cells survive.

## Conclusions

The imbalance of Th1/Th2 and Th17/Treg cells was related to the UCC stage, tumor size, lymph node metastases, and vasoinvasion. Immunological cell levels normalized sooner in CIN patients than in UCC patients after operations. Our findings suggested that the peripheral immunological cell levels reflect the patient’s condition. It might be useful for choosing therapeutic strategies and prognostication for uterine cervical cancer and CIN. Due to clinical constraints, the UCC patients we observed were all stage I–II patients who underwent surgeries. In the future, we will study patients with more advanced cervical cancer and explore if immunological cells have incredible changes in advanced cervical cancers.

## Data Availability

Data sharing is not applicable to this article as no datasets were generated or analysed during the current study.

## Abbreviations

UCC: Uterine cervical cancer
HPV: Human papillomavirus
CIN: Cervical intraepithelial neoplasia
FIGO: International Federation of Gynecology and Obstetrics
SCC: Squamous cell carcinoma
ADC: Adenocarcinoma
PMA: Phorbol myristate acetate
PBMCs: Peripheral blood mononuclear cells
S.D.: Standard deviation
